# Features analysis for identification of date and party hubs in protein interaction network of *Saccharomyces Cerevisiae*

**DOI:** 10.1186/1752-0509-4-172

**Published:** 2010-12-19

**Authors:** Mitra Mirzarezaee, Babak N Araabi, Mehdi Sadeghi

**Affiliations:** 1 Department of Computer Engineering, Islamic Azad University, Science and Research Branch, Tehran, Iran; 2 Control and Intelligent Processing Center of Excellence, School of Electrical and Computer Engineering, University of Tehran, Tehran, Iran; 3 School of Cognitive Sciences, Institute for Research in Fundamental Sciences, IPM, Tehran, Iran; 4 National Institute of Genetic Engineering and Biotechnology (NIGEB), Tehran, Iran; 5 School of Computer Sciences, Institute for Research in Fundamental Sciences, IPM, Tehran, Iran

## Abstract

**Background:**

It has been understood that biological networks have modular organizations which are the sources of their observed complexity. Analysis of networks and motifs has shown that two types of hubs, party hubs and date hubs, are responsible for this complexity. Party hubs are local coordinators because of their high co-expressions with their partners, whereas date hubs display low co-expressions and are assumed as global connectors. However there is no mutual agreement on these concepts in related literature with different studies reporting their results on different data sets. We investigated whether there is a relation between the biological features of *Saccharomyces Cerevisiae*'s proteins and their roles as non-hubs, intermediately connected, party hubs, and date hubs. We propose a classifier that separates these four classes.

**Results:**

We extracted different biological characteristics including amino acid sequences, domain contents, repeated domains, functional categories, biological processes, cellular compartments, disordered regions, and position specific scoring matrix from various sources. Several classifiers are examined and the best feature-sets based on average correct classification rate and correlation coefficients of the results are selected. We show that fusion of five feature-sets including domains, Position Specific Scoring Matrix-400, cellular compartments level one, and composition pairs with two and one gaps provide the best discrimination with an average correct classification rate of 77%.

**Conclusions:**

We study a variety of known biological feature-sets of the proteins and show that there is a relation between domains, Position Specific Scoring Matrix-400, cellular compartments level one, composition pairs with two and one gaps of *Saccharomyces Cerevisiae'*s proteins, and their roles in the protein interaction network as non-hubs, intermediately connected, party hubs and date hubs. This study also confirms the possibility of predicting non-hubs, party hubs and date hubs based on their biological features with acceptable accuracy. If such a hypothesis is correct for other species as well, similar methods can be applied to predict the roles of proteins in those species.

## Background

Proteins are important components of all living organisms. They are responsible for essential functions within cells. Most proteins perform their biological functions through interacting with other proteins [1]. Map of the whole physical protein interactions inside an organism forms a network called Protein Interaction Network (PIN). Although large-scale PINs have already been determined experimentally for several species; in general there is a lack of protein interaction data for many species, and the computational prediction of protein interactions are still among the most wanted solutions in protein bioinformatics [2]. These networks display scale-free topologies which are characterized by the power law distribution [3]. This means despite some negative remarks [4], in general a small fraction of proteins called hubs interact with many partners while majority of the proteins called non-hubs, interact with only a few others. Commonly proteins with more than eight interactions in the PINs are hubs and those with less than three interactions are non-hubs. Others are called intermediately connected (IC) [5].

Hubs have special properties that attracted great interests from both engineering and biology communities; random removal of non-hubs elicits no observable change in the structure of the network, whereas removal of hubs changes the structure of the network significantly [6]. Hubs are interesting drug targets for cancer research [7] also, it has been shown that there is a relationship between number of protein interactions and its sequence conservation, and cellular properties which can be used as identifiers for separating hubs from non-hubs [8,9].

Hubs of the PINs are classified as static or Party Hubs (PHs) which interact with most of their partners at the same time or Dynamic or Date Hubs (DHs) which change their interaction partners at different times or locations. Although the exact reasons for why date hubs change their partners are not clear yet, party and date hubs of the PINs are specified from the analysis of their gene co-expression profiles [10].

The study of PINs on a genome-wide scale is possible through advances in high-throughput experimental research. These experiments have generated large amounts of interaction data for several species including *S. Cerevisiae *[11-15], *Escherichia coli *[16], *Drosophila melanogaster *[17], *Caenorhabditid elegans *[18], and *Homo sapiens *[19,20]. The corresponding PINs are accessible through databases such as IntAct [21] and DIP [22].

Identification of hubs and non-hubs of a PIN is usually performed through network construction. For example hub object analyzer (Hubba) is a web-based service for identifying hubs in an interactome network generated from small- or large-scale experimental methods based on graph theory [23].

Computational approaches have also been developed to predict these networks using text-mining techniques [24,25], three dimensional structures [26-28], gene proximity [29,30], gene fusion events [31,32], gene co-expression [33-35], phylogenetic profiling [36], orthologous protein interactions [37], and identification of interacting protein domains [38-41]. The levels of intrinsic disorder, surface charge and domain distribution in hubs and non-hubs and differences in their functional domains are also studied [42]. Flexibility of hubs and comparison of date hubs and party hubs with non-hubs is evaluated using temperature factors [43]. However, no study has focused on separation of non-hubs from party hubs and date hubs.

Some researchers utilize sequences, biological data descriptors and Gene Ontology (GO) terms in identification of hubs and non-hubs of four different species [44-46]. However, they do not investigate the fusion of biological features, and their classifier is not capable of separating date hubs from party hubs.

Ekman *et al*. studied many different characteristics of non-hubs, party hubs and date hubs of *S. Cerevisiae *from domain features to protein age [5]. They showed that hubs should share certain common features that not only enable them to participate in several protein interactions, but also can be used for the theoretical identification of these hub proteins without prior knowledge of corresponding PINs.

The concepts of party hubs and date hubs are first proposed by Hen *et al*. based on gene co-expressions, using filtered yeast microarray data [10]. Based on another filtered yeast data set no evidence for coexistence of party hubs and date hubs is reported [47]. Agarwal *et al*. showed that small subsets of date hubs are important for network connectivity [48]. Party and date hubs are also studied using network motifs by Jin *et al*. [49]. They have found two types of hubs named motif party hubs (mPHs) and motif date hubs (mDHs). The authors showed that mPHs and mDHs display distinct biological functions. They also showed that hubs affect the topological structure of the network differently, that is deleting PHs has little influence on the network structure while deleting DHs breaks the network into many fragments. These observations emphasise the importance of identifying not only hubs from non-hubs, but also PHs from DHs. PHs and DHs control the architecture of the biological networks differently, and they are sources of biological complexity observed in the modular organization of such networks.

In the literature, there is no mutual agreement on the concepts of party and date hubs. In this paper we study the relation of biological features to the concepts of non-hubs, intermediately connected, party hubs, and date hubs. The relation between protein roles in a network and their biological characteristics may confirm the existence of party and date hubs.

This paper is focused on answering the following question: "*Which features should be used to better discriminate non-hubs, party hubs and date hubs in a PIN?*" A related sub-question is "*What classification methods more effectively discriminate these classes?*" In our experiments, we concentrate on *S. Cerevisiae's proteins *however, the proposed feature analysis methodology should be applicable to similar studies.

We formulated the problem as below: For a specific protein, assume *n *feature vectors from presumably *n *different sources

(1) X1=(x,11x12,...,x1k1),...,Xn=(xn1,xn2,...,xnkn),

where *i-*th feature vector consists of *k_i _*features, and *i = 1,2,...,n*. A classifier is a mapping from features space to one of the output values, *1 *to *4*, representing non-hubs, intermediately connected, party hubs, and date hubs of the PIN.

In this paper, different features, including amino acid sequences, domain contents, repeated domains, functional categories, biological process, cellular compartment, disordered regions, and Position Specific Scoring Matrix (PSSM), from various sources are extracted and studied. Some of these features have already been studied in identification of protein interactions or separating hubs from non-hubs [46], but they have not been used for discrimination between party hubs and date hubs of a PIN. However, in this work, we investigate all these features and some new ones. For example, evolutionary information in the form of PSSM has been used for prediction of protein secondary structure [50,51], and it has recently been used for predicting sub cellular localization of proteins [52,53]. However, in our study, PSSM has been used as a set of features for identification of four classes of proteins in the PIN of *S. Cerevisiae*.

## Results and Discussion

The Protein Interaction Networks (PINs) of many organisms are not fully determined yet. In the absence of complete PIN data, identification of non-hubs, party hubs, and date hubs based on their biological features becomes increasingly important. Drug design and study the modular organization and complexity of PINs are among the applications that benefit from such identification.

We focused on *S. Cerevisiae*, a species of budding yeast, in our experiments. *S. Cerevisiae's *identified PINs have approximately 16,000 to 40,000 interactions between its approximately 6,000 proteins. The data of *S. Cerevisiae's *non-hubs (NHs), Intermediately Connected (ICs), Party Hubs (PHs), and Date Hubs (DHs) was obtained from the supplementary material provided by Ekman *et al*. [5]. Table 1 shows the frequency of these four classes in *S. Cerevisiae *PIN.

**Table 1:** Distribution of four classes of proteins in S. Cerevisiae's PIN

Class Label	Number (Percentage) of Proteins
Non-Hub (NH)	4796 (81.4)
Intermediately Connected (IC)	575 (09.8)
Party Hub (PH)	195 (03.3)
Date Hub (DH)	322 (05.5)

Total	5,888 (100)

In our experiments, we examine seventeen different biological characteristics of proteins including amino acid sequences, domain contents, repeated domains, functional categories, biological processes, cellular compartments, disordered regions, and Position Specific Scoring Matrix (PSSM) as feature-sets. We classify Yeast proteins into four classes of NHs, ICs, PHs, and DHs based on each feature-set separately.

The available data is bisected to 70% and 30% portions which are used for training and testing purposes, respectively. Training and testing samples are selected from each of four classes, separately and randomly. Moreover, the training sets are partitioned into five parts for 5-fold cross validation.

### Input Feature Reduction Methods

We used different methods of feature reduction including Principle Component Analysis (PCA), Non-Linear PCA (NL-PCA), and Independent Component Analysis (ICA) to reduce the size of all seventeen input feature-sets. Our experimental results show that supervised PCA is the most effective method in feature reduction. The number of features in each feature-set shrinks to three combined features using supervised PCA.

### Base Classifiers

We used the seventeen reduced features-sets as the input for seventeen homogenous classifiers. We examined three base classifiers, including K-Nearest Neighbours (KNN), Bayes with Gaussian Probability Density Function (PDF), and Bayes with Mixture Density Model (MDM) PDF as base classifiers. The MDM is built with different number of PDFs for different classes. Bayes classifier with KNN and Parzen nonparametric estimation of PDF are examined as well. However, since protein labels are discrete, many neighbours of a protein have overlapping labels. Therefore, KNN and Parzen PDF estimators do not perform well in these cases. Our results confirm this intuition; therefore we concentrate on parametric PDF estimation methods.

A summary of the results from different base classifiers have been shown in Table 2. In these tables average CCR is the average of Correct Classification Rate for the four classes of NHs, ICs, PHs, and DHs based on their confusion matrix. Correlation coefficient of the actual and predicted labels is also computed for each method. The results show that in average, KNN is the least performing classifier compared to Bayes classifier with Gaussian and MD model as PDF estimators.

**Table 2:** Base classifiers comparison based on different feature-sets

*Feature*	*Average CCR (Corr. Coef.) %*		
	
	KNN	Bayes with Gaussian PDF	Bayes with MDM PDF
Amino Acid compositions	26.0 (11.5)	25.0 (50.0)	33.6 (15.0)
Dipeptides	31.7 (21.8)	31.5 (25.1)	43.9 (36.8)
PairsComp1Gap	31.5 (23.7)	31.6 (27.2)	43.0 (29.4)
PairsComp2Gaps	30.7 (21.0)	31.8 (25.7)	45.9 (34.4)
Haralick Features	26.9 (03.5)	26.6 (06.1)	26.0 (07.8)
48 physicochemical prop.	26.6 (10.3)	25.0 (50.0)	29.4 (13.5)
Biological Process level 1	31.8 (16.8)	27.8 (16.4)	34.3 (18.8)
Biological Process level 2	33.0 (22.2)	33.5 (18.0)	30.9 (14.1)
Cellular level 1	32.7 (25.8)	27.0 (19.0)	35.4 (29.5)
Cellular level 2	31.4 (28.5)	31.0 (20.2)	28.2 (14.5)
Functional Process level 1	30.0 (10.9)	27.3 (08.4)	27.6 (11.6)
Functional Process level 2	28.2 (15.8)	30.5 (17.9)	28.3 (15.7)
Domains	56.0 (60.0)	67.1 (58.9)	63.7 (55.5)
Repeated Domains	57.0 (59.3)	66.6 (57.7)	65.7 (57.1)
Disordered Regions	26.5 (07.5)	25.2 (-4.0)	27.2 (13.3)
PSSM-20	26.2 (08.4)	26.0 (08.4)	26.0 (09.3)
PSSM-400	37.0 (35.8)	42.6 (42.1)	54.1 (47.9)

### Feature Selection

We studied all the feature-sets and their classification results. These studies show that amino acid compositions and 48 physicochemical properties have sensitivity equal to one and specificity equal to zero. Zero specificity means that these feature-sets cannot discriminate true negative samples properly and they are not good candidates for separating protein classes.

Our experimental result on effectiveness of each classifier for each feature-set is shown in Table 2. Bayes classifier with Gaussian PDF work best for some of the feature-sets, while Bayes classifier with MDM PDF works better for others. That means the features such as domain, repeated domain, and Haralick have almost Gaussian distribution because the MD model does not improve the average CCR of the results. Most sequence related data performs better with the MDM and they are assumed to have a non-unimodal non-Gaussian probability density function. Based on the results shown in Table 2, six of feature categories can discriminate protein classes significantly better than the others in terms of average CCRs of classifiers and correlation coefficients of the results: repeated domains, domains, PSSM-400, cellular compartment level one, and amino acid composition pairs with two and one gaps.

### Feature Fusion

From the results of best base classifiers in the previous section, it is clear that most of the classifiers are weak learners. A good approach to deal with this problem is to fuse classifiers. If they are each expert in part of the studied subject, on the whole, it is expected that their fusion obtains better results. Here we opt an input feature fusion approach.

Another question is that how many of the input features should be fused. We first combined all the reduced input feature-sets and achieved an average CCR of 68.3%. That is a reasonable result because some of the tested features are not good candidates for separating our four classes of hubs and non-hubs and they reduce the final classification rate. We then used the following greedy forward selection algorithm to find the best fusion of feature-sets. At each step, one of the feature-sets with highest discrimination capability is added to the set of input features of the classifier, and if the average CCR and correlation coefficients are better than the previous combination, this feature-set is augmented to the base classifier's input feature-set. This process continuous until adding a new feature-set reduces the performance of the classification based on specified parameters.

Domain features alone have an average classification of 67% and a correlation coefficient of 58.9% and therefore they form our best feature-set. The final feature-sets that we chose are domains, PSSM-400, cellular compartment level one, and composition pairs with one and two gaps. We reached to an average CCR of 74.9% among four classes using Bayes classifier with Gaussian PDF. Results of step by step combination of input feature-sets are shown in Table 3. In this table, average CCR of each combination of feature-sets and their corresponding confusion matrix are shown. Adding any more features reduces the average CCR.

**Table 3:** Fusion of feature-sets with Gaussian Bayes classification

*Feature Fusion*	*Average CCR* *(Corr. Coef.)%*		*Confusion Matrix %*			
		
			NH	IC	PH	DH
All Features	68.3(62.3)	NH	**89.8**	06.7	01.0	02.6
		IC	34.4	**58.0**	03.2	04.5
		PH	14.9	13.5	**63.5**	08.1
		DH	22.1	14.7	01.1	**62.1**

Domains	67.0(58.9)	NH	**90.9**	03.4	01.9	03.8
		IC	36.3	**47.8**	04.5	11.5
		PH	18.9	02.7	**71.6**	06.8
		DH	20.0	08.4	13.7	**57.9**

Domain	66.8(58.0)	NH	**89.1**	03.5	03.5	03.8
RepDomains		IC	35.7	**47.1**	06.4	10.8
		PH	14.9	04.1	**73.0**	08.1
		DH	16.8	10.5	14.7	**57.9**

Domains	70.9(64.8)	NH	**90.2**	04.4	02.5	02.9
RepDomains PSSM-400		IC	35.0	**53.5**	06.4	05.1
		PH	10.8	05.4	**75.7**	08.1
		DH	14.7	07.4	13.7	**64.2**

Domains	71.7(65.4)	NH	**91.5**	04.0	01.5	03.1
PSSM-400		IC	35.0	**53.5**	04.5	07.0
		PH	12.2	06.8	**74.3**	06.8
		DH	16.4	06.3	09.5	**67.4**

Domains	74.0(67.1)	NH	**91.2**	04.2	01.6	03.0
PSSM-400		IC	31.8	**57.3**	05.1	05.7
Cellular1		PH	12.2	05.4	**75.7**	06.8
		DH	14.7	06.3	06.3	**72.6**

Domains	74.7(69.3)	NH	**91.7**	04.4	01.5	02.5
PSSM-400		IC	31.8	**58.0**	05.1	05.1
Cellular1		PH	09.5	08.1	**75.7**	06.8
CompPair2Gaps		DH	15.8	05.3	05.3	**73.7**

Domains	74.9(69.9)	NH	**92.1**	04.1	01.5	02.2
PSSM-400		IC	31.2	**59.2**	05.1	04.5
Cellular1		PH	13.5	06.8	**74.3**	05.4
CompPair2Gaps CompPair1Gap		DH	14.7	07.4	04.2	**73.7**

We have also tested the effects of using composition pairs with more than two gaps. These features have a slight effect on the results. We tested different combination of gapped composition pairs with other best selected features. The average CCR of the classifier changes by 0.8% using composition pairs with three and one gap together.

### Minimum Risk Classifiers as a Solution to Classify Unbalanced Data Sets

As shown in Table 2 and Table 3, in most cases classifiers do not work well when the number of available samples from each class is not the same and risk of correct hub classification is higher than that of non-hubs. To see if risk management improves the results, minimum risk version of the selected base classifiers are also examined. Results are shown in Table 4, where the following cost matrix is used:

(2) $$L=\left[\begin{array}{cccc} 0 & 0.1 & 0.2 & 0.2\\ 0.1 & 0 & 0.2 & 0.2\\ 0.9 & 0.9 & 0 & 0.2\\ 0.9 & 0.9 & 0.2 & 0 \end{array}\right].$$

**Table 4:** Minimum Risk extension of base classifiers on different feature-sets

*Feature*	*Average CCR (Corr. Coef.) %*		
	
	Min Risk KNN	Min Risk Bayes with Gaussian PDF	Min Risk Bayes with MDM PDF
Amino Acid compositions	30.2 (14.2)	29.4 (50.0)	28.4 (14.8)
Dipeptides	34.8 (26.2)	43.5 (25.1)	45.0 (34.0)
PairsComp1Gap	35.7 (26.9)	41.8 (27.2)	42.7 (29.0)
PairsComp2Gaps	37.0 (29.6)	41.1 (25.7)	45.6 (33.6)
Haralick Features	27.9 (07.6)	28.4 (06.1)	26.3 (06.1)
48 physicochemical prop.	29.5 (13.5)	29.0 (50.0)	29.0 (15.5)
Biological Process level 1	32.0 (19.2)	30.4 (16.3)	30.4 (14.9)
Biological Process level 2	34.1 (25.2)	35.7 (18.0)	37.7 (16.5)
Cellular level 1	34.2 (23.8)	35.4 (19.0)	36.6 (34.2)
Cellular level 2	35.8 (30.3)	34.0 (20.2)	36.6 (31.8)
Functional Process level 1	29.6 (17.3)	28.1 (08.4)	31.6 (14.5)
Functional Process level 2	29.6 (21.9)	33.2 (17.9)	30.6 (15.8)
Domains	60.5 (57.9)	67.5 (58.9)	67.8 (57.1)
Repeated Domains	59.6 (57.0)	67.4 (57.7)	63.9 (55.7)
Disordered Regions	27.0 (07.2)	26.1 (02.6)	27.7 (08.9)
PSSM-20	26.8 (06.1)	26.3 (08.4)	25.6 (01.7)
PSSM-400	43.8 (33.2)	49.8 (42.1)	54.0 (48.0)

The results with the selected best feature-sets and different maximum number of PDFs in the MDM are shown in Table 5. We achieved an average CCR of 73.7%, and a correlation coefficient of 72.1%.

**Table 5:** MDM Bayes classification with different number of PDFs for the best feature-set

*Number of PDFs*	*Average CCR* *(Corr. Coef.)%*
**2**	73.0 (70.5)
**3**	73.7 (70.7)
**4**	73.3 (72.1)
**5**	72.1 (70.6)

As the final step to improve the effectiveness of discrimination of party and date hubs, minimum risk versions of our best classifiers, Bayes with Gaussian and Mixture Density Model PDFs, are tested. As it is shown in Table 6, the Minimum Risk Bayes classifier with Gaussian PDF outperforms the MD model.

**Table 6:** Comparison of Minimum Risk classifiers on best fused features

***Classifier***	***Average CCR*** ***(Corr. Coef.)%***	***Confusion Matrix %***				
		
			**NH**	**IC**	**PH**	**DH**
	
Bayes with Gaussian PDF	77.0(69.4)	NH	**90.8**	03.7	01.8	03.6
		IC	29.3	**55.4**	06.4	08.9
		PH	08.1	04.0	**79.7**	08.1
		DH	07.4	05.3	05.3	**82.1**
	
Bayes with MDM PDF	74.4(69.6)	NH	**91.8**	03.1	02.6	02.5
		IC	31.2	**49.7**	08.3	10.8
		PH	10.8	0.0	**82.4**	06.8
		DH	11.6	03.2	11.6	**73.7**

A summary of the effectiveness of the both classifiers (Minimum Risk Bayes Gaussian and MDM) is shown in Table 6. Combination of the best feature-sets shows an average CCR of 77% among four classes, and correlation coefficient of 69.4% on the Minimum Risk Bayes classifier with Gaussian probability distribution.

A summary of the predicted labels in both classifiers (Minimum Risk Bayes Gaussian and MDM) are shown in Table 7.

**Table 7:** Predicted labels from both Min Risk Bayes classifiers with Gaussian and MDM models

	NHs	ICS	PHs	DHs	Average CCR	**Corr. Coef**.
Gaussian	1400	164	89	113	77.0	69.4
MDM	1384	111	127	144	74.4	69.6
True Labels	1440	157	74	95	-	-

**Figure 1:**
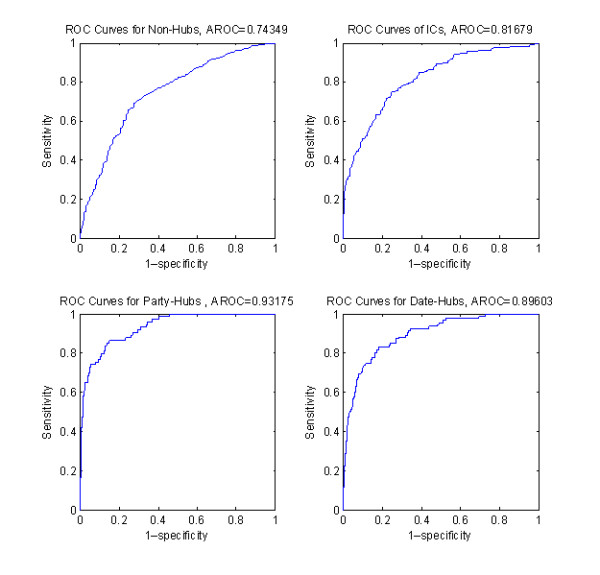
**Roc Curves and AROC values for Separating NHs, ICs, PHs, and DHs**.

We computed four metrics of specificity, sensitivity, Positive Predictive Value (PPV), and Negative Predictive Value (NPV) for multiclass classification. Results for the best classifier (Minimum Risk Bayes with Gaussian distribution) and the best feature-set combinations are shown in Table 8. These features can create an image of the final multi-class classifier effectiveness. Refer to the method section for details on their calculations. In addition to these metrics, Receiver Operator Characteristics (ROC) curves for binary separation of NHs, ICs, PHs, and DHs with their corresponding AROC values are shown in Figure 1.

**Table 8:** PH/DH/NH prediction results in S.Cerevisiae

	*Sensitivity%*	*Specificity%*	*PPV%*	*NPV%*
NHs vs. Others	90.8	81.9	95.7	66.9
ICs vs. Others	55.4	96.2	58.4	95.7
PHs vs. Others	79.6	97.6	59	99.1
DHs vs. Others	82.1	95.7	52	98.9
PH+DH vs. Others	87.5	93.6	59.2	98.6

### Analysis of the Results

The final feature-set seems reasonable since in eukaryotes an increased number of domain combinations are found. Also it is reasonable that a multi-domain protein can bind to many different proteins and the ratio of hub proteins which are multi-domain in the network is more than that of non-hub proteins. Since many repeated domains have binding functions, it is also reasonable that these proteins have more connectivity than single domain proteins and hub proteins with repeated domains are more probable than non-hubs in network. PSSMs represent the conserved motifs in protein families and because of the important roles of hub proteins and more connectivity of them, PSSM contains information for prediction of protein classes. Majority of the interactions occur between proteins in the same sub-cellular compartment and hub proteins, and their connected partners should be in the same compartment. Then cellular compartment information can help in discrimination of protein classes. It has been shown that the dipeptide compositions of proteins are important parameters for protein structure and have been used extensively to enhance the prediction quality for protein structural contents and cellular location [54,55].

## Conclusions

In this paper, we proposed a classification method for proteins of *Saccharomyces Cerevisiae*. These proteins were classified into four classes of non-hubs, intermediately connected, party hubs, and date hubs, based on their biological properties. Few works on identification of hubs in Protein Interaction Networks (PINs) have been done before, and to the best of our knowledge, none of them studied the discrimination of party hubs and date hubs.

Date hubs are important proteins of the network because they are the sources of observed dynamics. In this work, different protein feature categories including amino acid sequences, domain contents, repeated domains, functional categories, biological process, cellular compartment, disordered regions, and position specific scoring matrix were studied. Different methods of feature reduction including PCA, NL-PCA, and ICA were examined. We showed that supervised PCA was the most effective method. The reduced features from each category were utilized as the inputs to homogenous base classifiers. Different base classifiers including KNN, Bayes classifier with different parametric and non-parametric estimations of probability density function were investigated. Among different base classifiers, Bayes classifier with Gaussian distribution performs better with five feature categories of domains, PSSM-400, cellular compartment level one, and composition pairs with two and one gaps. The classifier results were compared based on average Correct Classification Rate (CCR) and correlation coefficients.

Combination of the best feature-sets showed an average CCR of 77% among four classes, and a correlation coefficient of 69.4% on the minimum risk Bayes classifier with Gaussian probability distribution.

We will further investigate other features such as 3 D structure of proteins in the future. The proposed feature analysis methodology can be applied to other species to predict unknown party and date hubs.

## Methods

### Extracted Protein Features

#### Amino Acid Sequences

Proteins are defined by their unique sequence of amino acid residues; these sequences are one of the well-known information sources for proteins. Amino acid sequences of *S. Cerevisiae *are obtained from NCBI http://www.ncbi.nlm.nih.gov on Jan. 2009. Six different groups of features are extracted from sequence data. In the sequel these features are briefly reviewed.

#### Amino Acid Compositions

Amino acid compositions (AAC) encapsulate the variable length protein sequences into fixed length -twenty dimensional- feature vectors [55]. AAC is the fraction of each amino acid in a protein sequence. The feature vector extracted from a protein sequence can be expressed by 20 coordinates, corresponding to 20 standard amino acids. The ACCs are calculated according to the following formula:

(3) $$comp(i)=R_{i}/N,i=1,2,...,20,$$

where *R_i _*is the number of amino-acid of type *i*, and *N *is the total number of amino-acids in a protein, that is, the length of that protein.

#### Dipeptide Compositions

Dipeptide Compositions (DC), amino acid composition pairs, are also computed from primary sequences [56]. DC is the occurrence frequencies of two consecutive residues in a protein. This feature vector extracted from protein sequences can be expressed by 400 coordinates. The advantage of dipeptides over amino acid composition is that they encapsulate information about the fraction of amino acids as well as their local order. Dipeptides are calculated according to the following formula:

(4) $$dpep(i)=D_{i}/\left(N-1\right),i=1,2,...,400,$$

where *D_i _*is the number of dipeptide of type *i *and *N *is the length of protein.

#### Amino Acid Composition Pairs with Gap

This feature calculates the fraction of some special patterns, like "*AxB*", inside the whole protein sequence, where *A *and *B *are certain known amino acids, while the gap "x" can be any amino acid. Up to four gaps are usually considered [56]. Amino acid composition pairs with *k *gaps are calculated according to the following formula:

(5) $$gapped\_dip_{k}(i)=E_{k}(i)/\left(N-1-k\right),i=1,2,...,400,$$

where *E_k_*(*i*) is the number of composition pairs with *k *gaps of type *i*, and *N *is the length of protein. This feature also has a fixed length of 400.

#### Co-occurrence Matrix and Features

One may notice the similarity between amino acid composition pairs with gaps and co-occurrence matrix in texture analysis [57]. This analogy encourages us to use those features defined on co-occurrence matrix to characterize *co-occurrence patterns *in amino acid sequences. The co-occurrence matrix in our study is a 20 by 20 matrix, where each column (or row) represents an amino acid. The elements of co-occurrence matrix come from (5). Some commonly used features are calculated from co-occurrence matrix, including: energy, correlation, inertia, entropy, inverse difference moment, sum average, sum variance, sum entropy, difference average, difference variance, difference entropy, and information measure of correlation. They are calculated as mentioned in [57].

#### Length of a Sequence

Proteins have different sequences with different lengths. Length of a protein is extracted as another feature.

#### Physicochemical Properties

Physicochemical properties of proteins like aromaticity, flexibility, and polarity are used as features. 48 features of this kind are introduced by Yu [58]; where for each feature the fraction of amino acids with that feature in a protein sequence is computed. For example aromatic property is the property of H, F, W, and Y amino acids. Now, for a protein, the total number of amino acids from this group to the length of protein is a measure of aromaticity.

#### Domain Contents and Repeated Domains

We extract domains of each protein from InterPro website http://www.ebi.ac.uk/interpro based on their UniPort codes. This data is extracted on June 2009. The total number of domains used in any of the *S. Cerevisiae*'s Proteins is 1816. An array of the length 1816 is constructed for each protein of the *S. Cerevisiae*, where if the specific domain exists in that protein the corresponding cell is set to one otherwise it is set to zero. Repeated domains are defined as two adjacent Domains from the same family. This feature-set is provided by Ekman *et al*. [5].

#### Functional Categories, Biological Process, and Cellular Compartments

The Gene Ontology(GO) has categorized the proteins of different organisms based on their functions, biological processes and cellular compartments in the cell [59-61]. These categories formed a graph based on which one can find these protein features with different levels of details. The file containing the whole GOs is obtained from GO website http://www.geneontology.org.

First, second and third level of functional categories, biological processes, and cellular compartment of each *S. Cerevisiae*'s proteins are extracted from July 2009 GO release. At the second level of the GO hierarchy, *S. Cerevisiae *proteins are classified into 19 different biological process, 8 different cellular compartments and 15 different molecular functions. In the third level, this grouping changes to 65 biological processes, 33 cellular compartments and 60 molecular function categories. These features are numerically coded in an array with the length of maximum number of available categories for each class. For each protein its biological process, cellular compartment and molecular function at level two and three of details are used as features.

#### Disordered Regions

Disordered regions -regions that lack a clear structure- are suggested to be important for flexible or rapidly reversible binding. To study whether disordered regions can separate four protein classes of interest, the relevant features are calculated as explained by Ekman *et al*. [5] using Dispred2 [62] at a 5% expected rate of false positives.

#### Position Specific Scoring Matrix

Position Specific Scoring Matrix (PSSM) is a commonly used representation of motifs (patterns) in biological sequences. They are derived from searching homologies in a protein database using multiple sequence alignment. This matrix of score values provides a weighted match to any amino acid symbol -a substring with fixed length. It has one row for each symbol of the amino acids, and one column for each position in the sequence [63].

In this research, the PSSM for each sequence is generated by PSI-BLAST search against 'nr' database using three iterations while e-value of cut off is 0.001 [64]. Two vectors with dimensions 20, and 400, namely PSSM-20, and PSSM-400 are generated from PSSM matrix. PSSM-20 is a simple composition of occurrences of each type of amino acids in the protein sequences of its homologues. In PSSM400, instead of one column for each amino acid residue, 20 values, corresponding to 20 standard amino acids types, are assumed. Hence, PSSM-400 is a vector of dimensions 20 by 20.

### Feature Selection

Three popular feature selection methods are used for dimensionality reduction of protein feature-sets including unsupervised and supervised Principle Component Analysis (PCA) [65], Non-linear PCA (NL-PCA) [66], and Independent Component Analysis (ICA) [67]. Each feature selection method is coupled with different classifiers, where results point at supervised PCA as the preferred feature selection method.

### Classification Methods

After applying feature reduction to separate feature-sets, different homogenous multi-class classifiers are applied to each feature-set. Here utilized classifiers are briefly introduced.

#### k-Nearest Neighbor Classifier

In *k*-Nearest Neighbor (KNN) classification [68], a majority voting among class labels of *k *nearest neighbors to a query protein determines the role of the protein. In this research, Euclidean distance is utilized as a distance measure. The best value for *k *is chosen by cross validation.

#### Bayes Classifier

In Bayesian decision theory the optimal class labels are chosen to minimize the probability of classification error [69]. In this framework we need to know a priori distribution of classes as well as class conditional Probability Density Functions (PDFs) for all classes. We opt two model based methods for PDF estimation.

#### I. Gaussian PDF Estimation

Perhaps the most commonly encountered PDF in practice is the Gaussian or Normal density function. We assumed the general multivariate form of normal density function, where mean and covariance of the PDF are estimated by means of training samples.

#### II. Mixture Density Model for PDF Estimation

Mixture Density Model (MDM) provides a more flexible model for PDF, by convex linear combination of simple component PDFs. The MDM is particularly good in modeling non-unimodal PDFs. The MDM can virtually approximate any arbitrary continuous PDF with the chosen accuracy, provided that sufficiently large number of component PDFs are combined and appropriate model parameters are estimated [70]. In this paper we used Gaussian PDF as component PDF.

#### Minimum Risk Classification Methods

When different errors in classification associate with different costs for the user, the probability of classification error is not the best criterion for classification. Minimum risk classifier is a variant for Bayes classifier designed to handle this situation [68]. A risk function is defined and minimized instead of error probability. In our classification problem PINs have many non-hub samples as compared to hubs, and correct identification of hubs is more important than non-hubs or intermediately connected proteins. As a result, minimization of risk function instead of error probability seems to be reasonable.

### Feature Fusion

The main goal of feature fusion is to increase the generalization capability of the classifiers. Each classifier is trained on a limited set of features. Thus pattern of classification error can be different from one classifier to another. Combination of input features of classifiers may hopefully results in a better performance [71]. In this problem we have fused the input feature-sets and studied which combination improves the performance of classification.

### Evaluation of the classifiers' Outputs

Four characteristics of specificity, sensitivity, Positive Predictive Value (PPV) and Negative Predictive Value (NPV) which are usually used for binary classifications are computed for the multi-class variant and used as a measure for evaluating classifiers' outputs according to the following formulas:

(6) $$\begin{array}{cc} Sensitivity=\frac{TP}{TP+FN} & PPV=\frac{TP}{TP+FP},\\ Specificity=\frac{TN}{TN+FP} & NPV=\frac{TN}{TN+FN} \end{array}$$

where TP, TN, FP, and FN stands for True Positive, False Positive, True Negative, and False Negative respectively.

## Authors' contributions

All authors participated in the design of the study and interpreting the results. MM implemented the method. The manuscript was written by MM and MS. All authors read and approved the final manuscript.
